# Gallic acid regulates primary root elongation via modulating auxin transport and signal transduction

**DOI:** 10.3389/fpls.2024.1464053

**Published:** 2024-09-02

**Authors:** Zilian Xu, Bing Yang, Jing Fan, Qiushi Yuan, Fu He, Hongwei Liang, Faju Chen, Wen Liu

**Affiliations:** Key Laboratory of Three Gorges Regional Plant Genetics & Germplasm Enhancement (CTGU)/Biotechnology Research Center, College of Biological and Pharmaceutical Sciences, China Three Gorges University, Yichang, Hubei, China

**Keywords:** Arabidopsis, gallic acid, auxin, primary root, root meristem

## Abstract

Gallic acid is an important secondary metabolite in plants, with great value in medicine, food, and chemical industry. However, whether and how this widely existing natural polyphenolic compound affects the growth and development of plants themselves remains elusive. In this study, we revealed that exogenous application of gallic acid has a dual effect on the elongation of primary root in Arabidopsis. While lower concentrations of gallic acid slightly stimulate primary root growth, excessive gallic acid profoundly reduces primary root length and root meristem size in a dose-dependent manner, probably via suppressing cell division in root meristem as indicated by *CYCB1;1::GUS*. Moreover, as suggested by the *DR5::GFP* line analysis and confirmed by the LC-MS assay, auxin contents in root tips were dramatically decreased upon excessive gallic acid treatment. Additional application of IAA partially rescued the shortened primary root and root meristem upon excessive gallic acid treatment, suggesting that auxin is required for excessive gallic acid-caused root growth inhibition. Then, we further revealed that excessive gallic acid down-regulated the expression of auxin transporters *PIN1*, *PIN2*, *PIN3*, and *PIN7*, and triple mutant *pin1 pin3 pin7* exhibited a reduced sensitivity to gallic acid treatment. Meanwhile, excessive gallic acid decreased the degradation of AXR3/IAA17 protein as revealed by *HS::AXR3NT-GUS* reporter line. Auxin signaling mutant *tir1 afb2 afb3* and *axr3-3* were also less sensitive to excessive gallic acid treatment in terms of primary root length and root meristem size. Taken together, these findings suggested that excessive gallic acid inhibits primary root growth by modulating auxin transport and signaling in Arabidopsis.

## Introduction

Gallic acid (3,4,5-Trihydroxybenzoic acid) is a natural polyphenolic compound, widely present in Chinese medicinal materials such as Chinese gallnuts, as well as fresh fruits such as pomegranates and mangoes. Modern medical research reported that gallic acid has multiple effects including antioxidant, anti-tumor, anti-inflammatory, and antibacterial, and thus elevated the medicinal value of those medicinal plants rich in this substance (Bharathi et al., 2023; Lü et al., 2023; Ni et al., 2024). Besides, as an important chemical raw material, its derivatives are also widely used in polymer materials (Al Zahrani et al., 2020). In certain extreme situations, gallic acid and its derivatives may accumulate to a high level in plant tissues. For instance, in the Chinese gallnuts, a kind of “insect gall” on the leaf wings of *Rhus chinensis* induced by gall-forming aphid (Takeda et al., 2019), gallotannins content accounts for up to 70% of total dry weight (Chen et al., 2018). So far, whether and how gallic acid participates in regulating plant growth and development remains largely unknown.

The development of plant root system is highly plastic (Satbhai et al., 2015). Through integrating endogenous and exogenous signals, plants can adjust their growth and development to adapt to various stresses. Previously, some of the natural and chemosynthetic polyphenolic compounds have been assayed to evaluate the effects of oxidation-reduction (redox) status on modulating the development of plant root system. For instance, catechol, a major constituent in smoke, affects plant root development through reactive oxygen species (ROS)-mediated redox signaling (Wang et al., 2017). Glutathione (GSH) is critical for maintaining and controlling cellular redox status, and inhibition of its synthesis by buthionine sulphoximine (BSO) resulted in reduced primary root elongation in Arabidopsis (Koprivova et al., 2010). Hydrogen peroxide (H_2_O_2_), one of the main forms of ROS in plants, activates mitogen-activated protein kinase 6 (MAPK6) to modulate nitric oxide production and signal transduction during Arabidopsis root development (Wang et al., 2010). In addition, biotic and abiotic stresses could also modulate plant root growth via regulating the endogenous redox status, since ROS burst and disruption of intracellular redox balance are common events under stresses. Jiang et al. (2016) reported that the cellular redox status differs between root meristem and elongation zone, and salt stress influences the redox status to interfere root meristem maintenance. In summary, polyphenolic compounds, including gallic acid, with their potential to regulate endogenous redox balance, likely play a role in modulating plant root growth and development. This study specifically explores how gallic acid affects primary root elongation in Arabidopsis.

Plant hormones are fundamental signaling molecules, which integrate endogenous and exogenous cues to coordinate the growth and development of plant organs (Zhang et al., 2023). Among them, auxin is commonly thought to play an essential role in root system architecture (Fukui and Hayashi, 2018). A series of tightly regulated processes control auxin biosynthesis, transport, and signal transduction, which function together to regulate auxin-mediated plant growth and development (Roychoudhry and Kepinski, 2022). An auxin maximum in root tips is maintained through polar auxin transport (PAT) during the early developmental stage in Arabidopsis (Blilou et al., 2005). PAT requires a series of membrane-bound auxin transport facilitators, including influx AUXIN RESISTANT 1/LIKE AUX1 (AUX1/LAX) and efflux PIN-FORMED (PIN) proteins, which exhibit distinctive but overlapped expression pattern in roots and functions redundantly to regulate primary root elongation and root meristem size (Blilou et al., 2005; Liu et al., 2015; Sauer and Kleine-Vehn, 2019). Meanwhile, auxin perception requires its specific receptors TRANSPORT INHIBITOR RESPONSE1 (TIR1)/AUXIN SIGNALING F-BOX (AFB), followed by degradation of the Aux/IAA proteins, release of auxin response factors (ARFs), and activation of the expression of auxin-responsive genes (Yu et al., 2022). The loss-of-function mutant *tir1 afb2 afb3* and gain-of-function mutant *axr3-3* exhibited a shortened primary roots and reduced root meristem size (Dharmasiri et al., 2005), indicating that auxin signaling transduction plays a key role in regulating primary root growth and development in Arabidopsis.

In this study, we found that exogenous application of gallic acid dramatically inhibited primary root elongation and reduced root meristem size in Arabidopsis. Gallic acid could decrease auxin contents in root tips by down-regulating the expression of *PINs* auxin transporter and suppress auxin signaling by promoting the stability of AXR3/IAA17 protein. In addition, the *pin1 pin3 pin7*, *tir1 afb2 afb3*, and *axr3-3* mutants exhibited reduced sensitivity to gallic acid treatment in terms of primary root length and root meristem size, suggesting the possible involvement of auxin transport and signaling in this progress.

## Materials and methods

### Plant materials and growth conditions

Arabidopsis ecotype Columbia (Col-0) was used as wild-type plants in this study. The following transgenic and mutant lines were described previously (Liu et al., 2015): *DR5::GFP*, *PIN1::PIN1-GFP*, *PIN2::PIN2-GFP, PIN3::PIN3-GFP*, *PIN7::PIN7-GFP*, *CYCB1;1::GUS*, *pin1 pin3 pin7*, *tir1 afb2 afb3*, *HS::AXR3NT-GUS* (CS9571), *pin2* (CS8058), *pin3* (CS9364), *pin7* (CS9367), *axr3/iaa17* (SALK_065697 and SALK_011820), and *axr3-3* (CS57505). The Arabidopsis seeds were surface-sterilized with 5% (w/v) bleach containing 0.1% (v/v) Triton X-100 for 5 min, and washed three times with sterile water before incubating for 3 d at 4°C in the dark. Thereafter, the seeds were planted onto one-half strength MS solid medium (Sigma-Aldrich) containing 0.8% (w/v) agar and 1% (w/v) sucrose, and then placed in a growth chamber maintained at 23°C, under long-day conditions (16-h-light/8-h-dark cycle) at a light intensity of 80 mmol photons m^-2^ s^-1^. For *HS::AXR3NT-GUS* assay, 5-day-old seedlings grown on normal one-half strength MS plates were transferred to liquid one-half strength MS medium and then heat-shocked at 37°C in the dark for 2 hours. Then, gallic acid stock was quickly added into the above liquid one-half strength MS medium to a final concentration of 0 μM and 500 μM. Thereafter, heat-shocked *HS::AXR3NT-GUS* seedlings were separated into liquid one-half strength MS medium containing 0 μM or 500 μM gallic acid and kept in the light incubator at 23°C, and samples were taken for GUS staining at 0 min, 60 min, and 120 min.

### Measurement of primary root length and root meristem size

The powder of gallic acid (Macklin, G823163, CAS#149-91-7) was dissolved in sterile water to produce 50 mM stock solutions, and then diluted to different concentrations in one-half strength MS solid medium. The primary root length and root meristem size were measured according to published methods (Dello Ioio et al., 2007; Liu et al., 2015). Briefly, seeds were planted onto plates supplemented with various components and grown in a vertical position for indicated days. For measurement of the primary root length and root meristem size, different concentrations of 0 μM, 0.5 μM, 1 μM, 10 μM, 50 μM, 100 μM, 200 μM, and 500 μM gallic acid were applied and a time course of 3 days, 5 days, 7 days, 9 days, and 11 days were monitored. For subsequent assays on different marker lines and mutants, the concentrations of 0 μM, 100 μM, 200 μM, and 500 μM gallic acid were used for 5 days and 7 days. In addition, 200 μM gallic acid plus 0.1 nM or 0.5 nM IAA (for IAA recovery assay) and 200 μM gallic acid plus 2 μM NPA (for NPA assay) were used for 5 days and 7 days. Thereafter, digital images of seedlings were captured and primary root lengths were measured by ImageJ software at indicated days after germination (dag). For measurement of root meristem size, root tips were excised and mounted immediately on glass slides with clearing solution (50 g of chloral hydrate, 15 mL of water, and 10 mL of glycerol), and then examined using an inverted fluorescence microscope (Leica DMi8). For measurement of primary root length, at least 30 individual seedlings were analyzed for each treatment or genotype. For measurement of root meristem size, at least 25 individual seedlings were analyzed for each treatment or genotype.

### GUS staining assays

GUS histochemical staining was carried out as previously described (Liu et al., 2015). In brief, seedlings of *CYCB1;1::GUS*, *QC25::GUS*, *QC46::GUS*, and *HS::AXR3NT-GUS* reporter lines were incubated at 37°C in GUS staining buffer (100 mM sodium phosphate buffer at pH 7.5, 10.0 mM EDTA, 0.5 mM potassium ferricyanide, 0.5 mM potassium ferrocyanide, and 0.1% [v/v] Triton X-100) supplemented with 1 mM 5-bromo chloro-3-indolyl-b-D-glucuronide. The incubation time was determined according to distinct marker lines: 4 h for *CYCB1;1::GUS*, 12 h for *QC25::GUS* and *QC46::GUS*, and 8 h for *HS::AXR3NT-GUS*. The stained seedlings were then kept in 70% ethanol to remove the chlorophyll, and the decolorized seedlings were observed and photographed under the bright field of an inverted fluorescence microscope (Leica DMi8).

### Quantification of IAA and gallic acid contents

Endogenous IAA and gallic acid contents were quantified by liquid chromatography-mass spectrometry (LC-MS). Briefly, wild-type Arabidopsis Col-0 was grown continuously for 5 days and 7 days on one-half strength MS solid medium supplemented with 0 μM and 200 μM gallic acid. For quantification of IAA, total roots were isolated and collected by rapidly freezing in liquid nitrogen. Roots of approximately 200 mg fresh weight were ground in liquid nitrogen to a fine powder, extracted with 80% (v/v) methanol, purified with a Poly-Sery MAX SPE Cartridge (CNW, Germany), and eluted with 0.5% formic acid (v/v) in methanol. The eluate was further fried under nitrogen, reconstituted in methanol, and injected into a high-performance liquid chromatography (HPLC)-MS/MS system consisting of an Agilent 1290 HPLC system (Aglient Company, USA) and a SCIEX-6500 Qtrap MS/MS tandem mass spectrometer (AB SCIEX Company, USA). For quantification of gallic acid, seedlings of approximately 100 mg fresh weight were added into 1 mL 50% methanol aqueous solution, ultrasonically extracted in ice water bath, centrifugated at 4 °C and 12000 g for 5 minutes, and then the supernatant was filtered by 0.22 μm organic phase filter membrane and injected into a HPLC-MS/MS system consisting of a LC-20AD HPLC system (Shimadzu Company, Japan) and a SCIEX-5500 Qtrap MS/MS tandem mass spectrometer (AB SCIEX Company, USA).Quantification of IAA and gallic acid contents was performed at Wuhan ProNets Testing Technology Co, Ltd. For each sample, three independent biological replicates were carried out.

### Detection and quantification of GFP fluorescence

The seeds of *DR5::GFP*, *PIN1::PIN1-GFP*, *PIN2::PIN2-GFP*, *PIN3::PIN3-GFP*, and *PIN7::PIN7-GFP* marker lines were germinated on one-half-strength MS solid medium containing different concentrations of gallic acid for the indicated time, and the seedlings of GFP lines were mounted onto microscope slides with sterile water for immediate observation. GFP fluorescence images in root tips were captured by an inverted fluorescence microscope (Leica DMi8). The GFP fluorescence intensities were quantitatively analyzed by Photoshop CS5 software. At least 20 root tips were taken and measured for each treatment and genotype.

### RNA isolation and quantitative real-time PCR

Total RNA was extracted from Arabidopsis roots using FastPure^®^ Universal Plant Total RNA Isolation Kit (Vazyme, RC411-01) according to the manufacturer’s instructions as previously described (Liu et al., 2015). To remove the contaminating DNA, DNase I (Vazyme, EN401-01) was used to digest the isolated RNA samples. Subsequently, reverse transcription was performed using BeyoRTTMII First-stand cDNA Synthesis Kit (Beyotime, D7168M). qRT-PCR was carried out on a Bio-Rad CFX96 apparatus with TB Green^®^ Premix Ex TaqTM (Tli RNaseH Plus) (Takara, RR420A). PCR reaction was carried out under a two-step procedure: 3-min incubation at 95°C for complete denaturation, followed by 45 cycles of 95°C for 15 s and 60°C for 45 s, ended by a melt curve from 60°C to 95°C. Based on analysis with geNorm software, *eukaryotic Initiation Factor 4* (*eIF4*) was selected from eight candidate reference genes as the most stable one in our conditions. At least three independent biological replicates and three technical repetitions were performed for each data. The primer sequences for qRT-PCR were listed in **Supplementary Table S1**.

### Statistical analysis

At least three biological repetitions were carried out for each experiment. For multiple comparisons, the SPSS 26.0 software was used to confirm the normality and homogeneity of variances of the data and perform one way ANOVA, and then significance of differences between multiple samples was determined by Woller-Duncan test (*P*<0.05). For paired comparisons, significance of differences was determined by Student’s *t* test (*P<0.05, **P<0.01, ***P<0.001) via the SPSS 26.0 software.

## Results

### Gallic acid reduces primary root length and root meristem size in Arabidopsis

**Figure 1:**
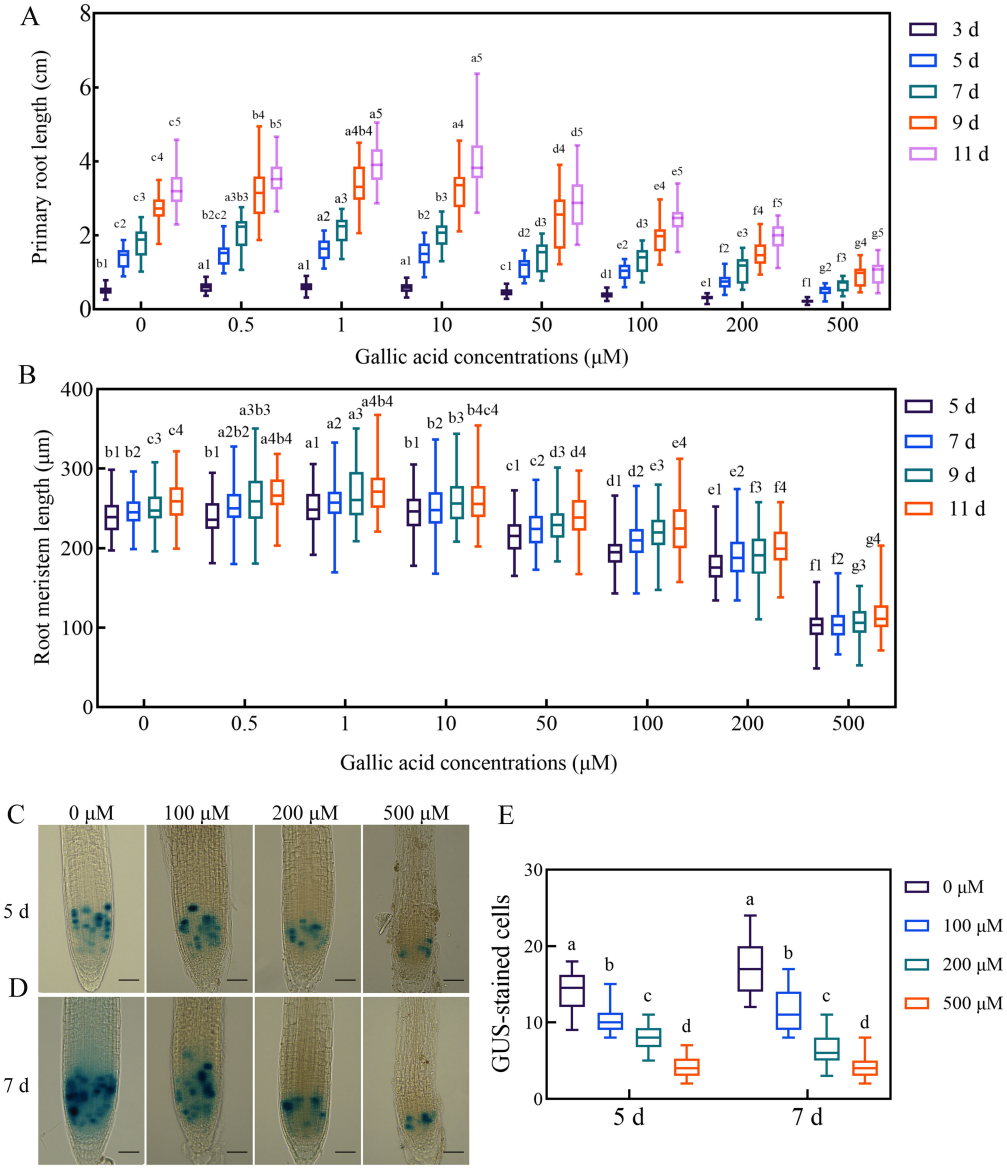
Gallic acid regulates primary root development by affecting the cell division activity in meristematic zone. **(A, B)** Time course of the root length **(A)** and the meristem size **(B)** in wild-type plants treated with different concentrations of gallic acid. Identical numbers following letters represent statistical data on the same day, and different letters indicate significantly different values (Woller-Duncan test, *P*<0.05). **(C, D)** GUS histochemical staining of *CYCB1;1::GUS* reporter lines treated with 0 μM, 100 μM, 200 μM, and 500 μM gallic acid for 5 d **(C)** and 7 d **(D)**. Bars = 50 μm. **(E)** Number of GUS-stained cells in root tips of *CYCB1;1::GUS* seedlings. Different letters indicate significantly different values (Woller-Duncan test, *P*<0.05).

To establish the relationship between gallic acid and root development in Arabidopsis, we firstly examined the effects of different concentrations of exogenous gallic acid on primary root elongation. The seeds of Col-0 wild-type Arabidopsis were surface-sterilized, planted on one-half strength MS plates supplemented with 0.5 μM, 1 μM, 10 μM, 50 μM, 100 μM, 200 μM, and 500 μM gallic acid, and then placed in a growth chamber. We found that the seed germination of wild-type plants was not affected by exogenous application of gallic acid. Thereafter, the lengths of primary roots and root meristems were measured at 3 days, 5 days, 7 days, 9 days, and 11 days post germination (dpg), respectively. Our data showed that exogenous application of lower concentrations of gallic acid (0.5 μM, 1 μM, and 10 μM) could slightly but significantly promote the growth of primary roots (**Figure 1A**, **Supplementary Figure S1**). For instance, at 5 dpg and 7 dpg, the primary root length increased by a maximum of 12.98% and 14.69% under 1 μM gallic acid treatment (**Figure 1A**, **Supplementary Figure S1**). On the contrary, under higher concentrations of 50 μM, 100 μM, 200 μM, and 500 μM gallic acid, the primary root length was dramatically decreased to 80.06%, 72.60%, 52.32%, 37.01% of control at 5 dpg and to 79.65%, 73.80%, 57.86%, 35.37% of control at 7dpg (**Figure 1A**, **Supplementary Figure S1**). Taken together, our results suggested that exogenous application of higher concentrations of gallic acid reduces primary root elongation in a dose-dependent manner in Arabidopsis.

Previous researches have revealed that primary root elongation is largely dependent on the cell division activity of root apical meristem (Blilou et al., 2005; Sabatini et al., 2003). To further figure out how gallic acid suppresses root growth, we monitored the changes in root meristem sizes. Our results indicated that in accordance with the changes in primary root lengths, the root meristem sizes were also enlarged slightly under lower concentrations of gallic acid, but reduced tremendously under higher concentrations in a dose-dependent manner (**Figure 1B**). For example, the root meristem sizes of wild-type plants under 0.5 μM, 1 μM, 10 μM, 50 μM, 100 μM, 200 μM, and 500 μM gallic acid treatment were 100.37%, 104.47%, 100.50%, 89.40%, 81.14%, 74.92%, 33.81% of control at 5 dpg, and 102.06%, 104.32%, 101.46%, 90.78%, 86.35%, 77.77%, 41.57% of control at 7 dpg. To further investigate the possible effects of excessive gallic acid accumulation in plants, highest concentrations (100 μM, 200 μM, or 500 μM) of gallic acid were used in subsequent experiments. The early stage of post-germination development is critical for plant root growth since the root meristem enlarged fast (Moubayidin et al., 2010). Since the primary root length is too short and the differences between wild type and mutants are barely detectable at 3 dpg, 5 dpg and 7 dpg were subsequently chosen for further analysis.

Previous study indicated that gallic acid is detectable in the root exudates of Arabidopsis (Narasimhan et al., 2003), but its native concentration in tissues of Arabidopsis remains unknown. To figure out this, we monitored the endogenous gallic acid content in Arabidopsis by LC-MS. Our data showed that the gallic acid contents in wild-type seedlings at 5 dpg and 7 dpg were about 0.069 ± 0.001 μg/g and 0.129 ± 0.002 μg/g, while its amounts increased to 9.019 ± 0.107 μg/g and 8.351 ± 0.024 μg/g when grown on the medium containing 200 μM gallic acid (**Supplementary Figure S2**). These data suggested that gallic acid is natively existing in Arabidopsis and exogenous application of gallic acid increased its endogenous level.

In addition, we tested the effects of 3,4-dihydroxybenzoic acid on primary root elongation, and found that it had a slightly weaker inhibitory effect on primary root elongation as gallic acid (3,4,5-trihydroxybenzoic acid, **Figures 1A, B**, **Supplementary Figure S3**). These results indicated that polyphenols like gallic acid and its analogs may have similar effects on primary root elongation in Arabidopsis.

To further clarify how gallic acid suppresses root meristem size, we firstly investigated the cell division activity in root apical meristem by *CYCB1;1::GUS* reporter line. Histochemical staining of *CYCB1;1::GUS* showed that compared with control, GUS-stained cells in root meristem were significantly decreased when treated with 100 μM, 200 μM, and 500 μM gallic acid both at 5 dpg (**Figures 1C, E**) and 7 dpg (**Figures 1D, E**), suggesting that gallic acid could suppress cell division in root meristem. Then, we evaluated the role of gallic acid in stem cell niche activity by using both *QC25::GUS* and *QC46::GUS* lines (Sabatini et al., 2003). However, GUS staining was observed in the roots of both the treated and control plants in *QC25::GUS* (**Supplementary Figure S4**) and *QC46::GUS* (**Supplementary Figure S5**), suggesting that stem cell niche activity may not be responsible for the reduced root meristem under gallic acid treatment.

### Gallic acid regulates root development via reducing auxin accumulation in root tips

**Figure 2:**
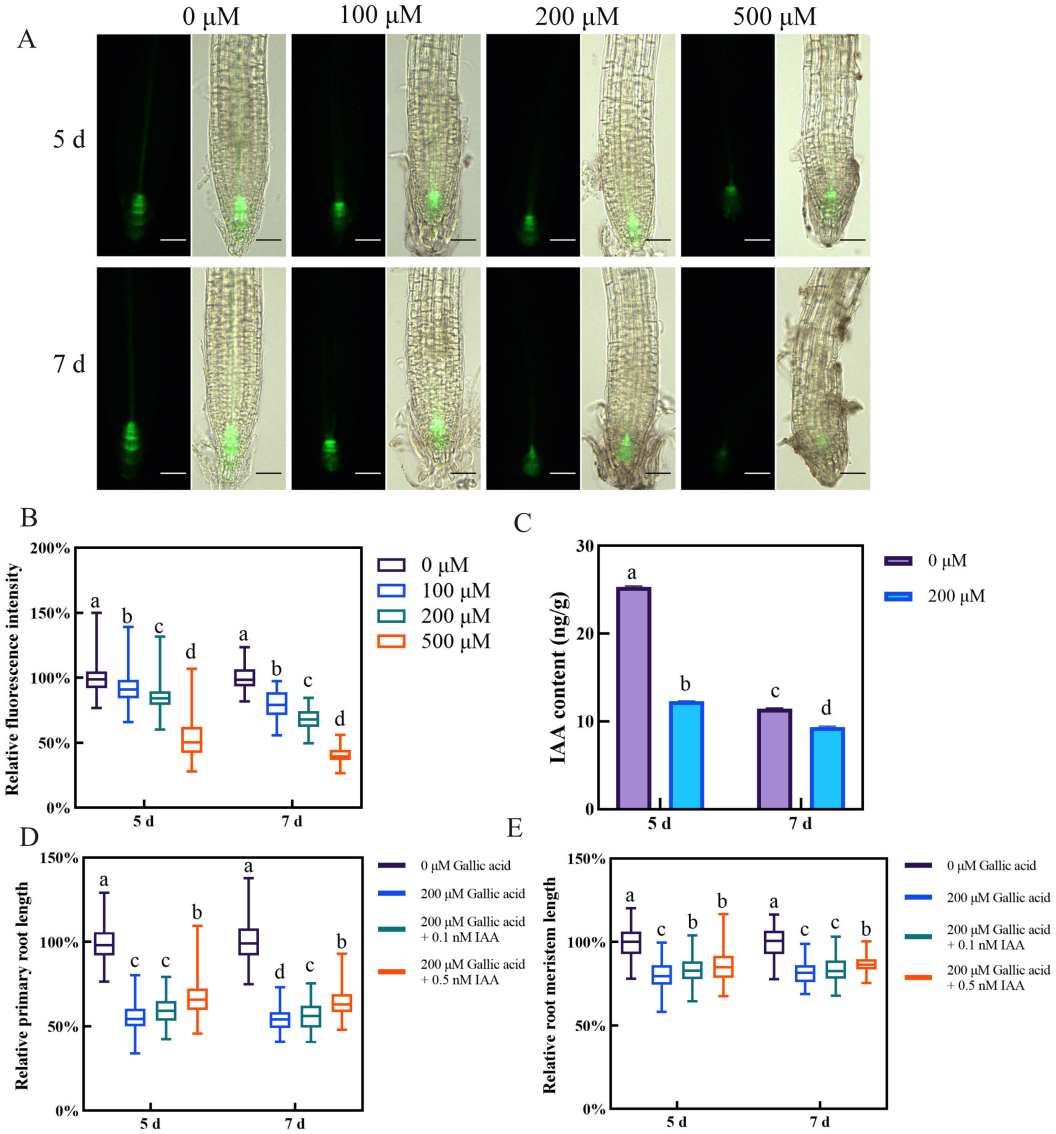
Gallic acid reduces auxin accumulation in roots, leading to primary root growth inhibition. **(A)** Fluorescence images of *DR5::GFP* reporter line grown for 5 d and 7 d on one-half strength MS plates supplemented with 0 μM, 100 μM, 200 μM, and 500 μM gallic acid. Bars=50 μm. **(B)** Quantitative analysis of *DR5::GFP* fluorescence intensity in A. Fluorescence intensity in untreated controls was defined as 100%. **(C)** Auxin contents in roots treated with 0 μM and 200 μM gallic acid for 5 d and 7 d as determined by LC-MS. **(D, E)** The relative lengths of primary roots **(D)** and meristem zones **(E)** in wild-type plants treated without or with 200 μM gallic acid plus 0 nM, 0.1 nM, or 0.5 nM IAA for 5 d and 7 d. Error bars represent SE. Different letters indicate significantly different values (Woller-Duncan test, *P*<0.05).

The plant hormone auxin plays a pivotal role in regulating root growth and development in Arabidopsis (Adamowski and Friml, 2015). The defective root meristem patterning observed upon gallic acid treatment raised the question of whether auxin content or auxin signaling is affected by excessive gallic acid. Hence, we first monitored the auxin response in root tips using the auxin-responsive *DR5::GFP* marker line (Friml et al., 2003). The fluorescence intensities of *DR5::GFP* in those root tips grown under 100 μM, 200 μM, and 500 μM gallic acid were dramatically lower than that in control roots (**Figures 2A, B**, **Supplementary Figure S6**). Additionally, quantitative analysis of endogenous IAA contents using liquid chromatography-mass spectrometry (LC-MS) also revealed that IAA levels in roots were significantly reduced when treated with 200 μM gallic acid (**Figure 2C**). Based on these results, we demonstrated that excessive gallic acid reduced auxin accumulation in root tips.

Next, we wanted to know whether decreased auxin accumulation is responsible for excessive gallic acid-induced inhibition of primary root growth. For this, the seeds of Col-0 wild-type Arabidopsis were grown on one-half strength MS plates without or with 200 μM gallic acid plus 0 nM, 0.1 nM, or 0.5 nM IAA, and the primary root length and root meristem size were measured at 5 dpg and 7 dpg. Although the primary root length and root meristem size were both reduced upon 200 μM gallic acid treatment, additional application of IAA led to a longer primary root length and enlarged root meristem size in roots subjected to 200 μM gallic acid treatment (**Figures 2D, E**), indicating that exogenous application of IAA could at least partially rescue the root growth inhibition caused by excessive gallic acid. In addition, we tested the effects of NAA in growth-rescue experiment, as it penetrates better through the membranes by passive diffusion. We found that NAA also rescued gallic acid-caused primary root inhibition with a maximum recovery effect of 14% in primary root length and 10% in root meristem size (**Supplementary Figure S7**).

### Gallic acid inhibits the expression of auxin transporters *PINs*

**Figure 3:**
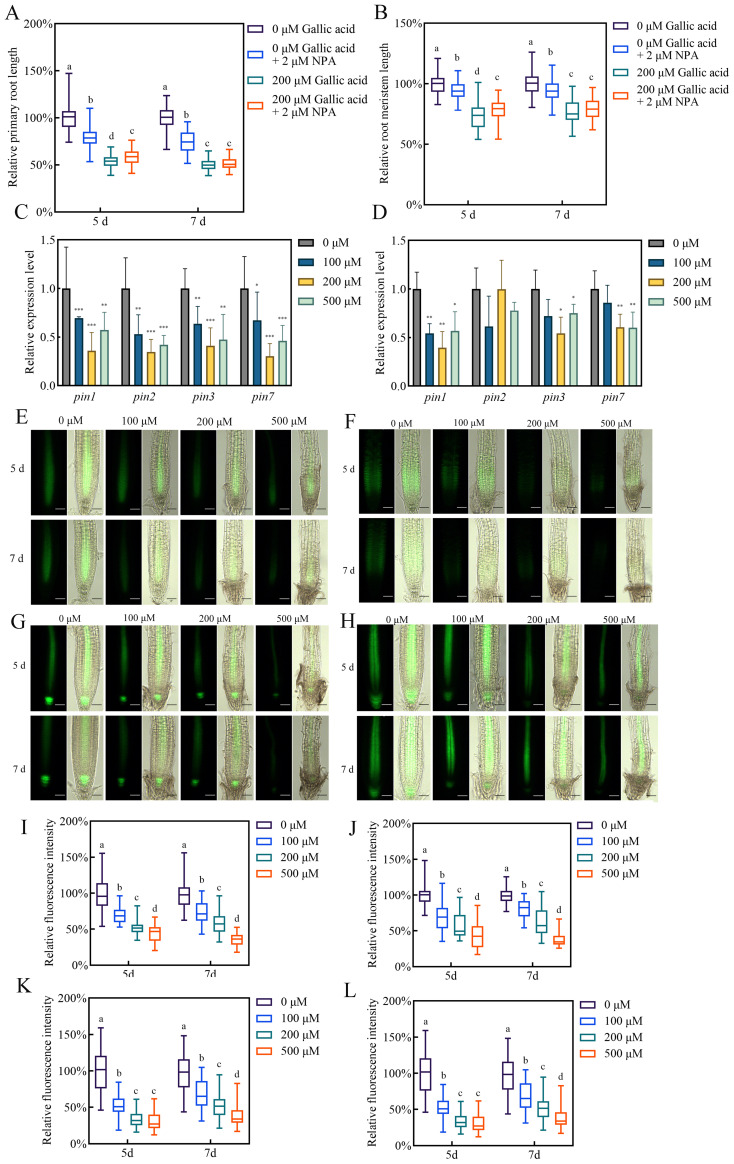
Gallic acid down-regulates the expression of auxin effluxes *PIN1*, *PIN2*, *PIN3*, and *PIN7*. **(A, B)** The relative lengths of primary roots **(A)** and meristem zones **(B)** in wild-type plants treated without or with 200 μM gallic acid plus 0 μM or 2 μM NPA for 5 d and 7 d **(C, D)** The relative expression levels of *PIN1*, *PIN2*, *PIN3*, and *PIN7* in wild-type roots treated with 0 μM, 100 μM, 200 μM, and 500 μM gallic acid for 5 d **(C, D)**. The expression levels of the indicated genes in untreated roots were set to 100%. **(E–H)** Images of *PIN1::PIN1-GFP* **(E)**, *PIN2::PIN2-GFP* **(F)**, *PIN3::PIN3-GFP* **(G)**, and *PIN7::PIN7-GFP* **(H)** reporter lines treated with 0 μM, 100 μM, 200 μM, and 500 μM gallic acid for 5 d and 7 d Bars=50 μm. **(I–L)** Quantitative analysis of *PIN1::PIN1-GFP* **(I)**, *PIN2::PIN2-GFP* **(J)**, *PIN3::PIN3-GFP* **(K)**, and *PIN7::PIN7-GFP* **(L)** fluorescence intensity. Fluorescence intensity in untreated controls was defined as 100%. Different letters indicate significantly different values (Woller-Duncan test, *P*<0.05) in Figure **(A, B, I–L)**. Error bars represent SE, and asterisks indicate significant differences with respect to the corresponding control (Student’s *t* test, *P<0.05, **P<0.01, ***P<0.001) in **(C, D)**.

Polar auxin transport from shoot to root contributes to auxin accumulation in Arabidopsis root apex as well as maintaining sustained primary root growth (Blilou et al., 2005; Su et al., 2022). The reduced auxin contents in root tips may be due to changes in PAT. Hence, we measured the primary root lengths and root meristem sizes in wild-type plants treated with 200 μM gallic acid in the presence or absence of 2 μM naphthylphthalamic acid (NPA), a widely-used auxin transport inhibitor (Su et al., 2022). Whereas treatment with NPA or gallic acid alone inhibited primary root growth, NPA could not further reduce the primary root length (**Figure 3A**) and root meristem length (**Figure 3B**) by the presence of gallic acid, indicating that PAT is essential for excessive gallic acid-modulated inhibition of root development.

The auxin carriers-mediated PAT is responsible for auxin accumulation and distribution in root tips (Adamowski and Friml, 2015; Petrásek and Friml, 2009). The reduced auxin accumulation observed in roots in response to excessive gallic acid may be due to the down-regulation of the expression of auxin transporters *PIN1*, *PIN2, PIN3*, and *PIN7*, which were previously reported to be critical efflux for long-term shoot-to-root auxin transport (Petrásek and Friml, 2009; Yang et al., 2022). Our qRT-PCR results showed that the transcript levels of *PIN* genes were down-regulated to various degrees in roots under excessive gallic acid treatment (**Figures 3C, D**). Moreover, we examined the changes of PIN proteins by using the reporter lines *PIN1::PIN1-GFP*, *PIN2::PIN2-GFP, PIN3::PIN3-GFP*, and *PIN7::PIN7-GFP*. Detection of *PIN-GFP* fluorescence showed that the protein levels of PIN1 (**Figures 3E, I**), PIN2 (**Figures 3F, J**), PIN3 (**Figures 3G, K**), and PIN7 (**Figures 3H, L**) were also reduced by excessive gallic acid treatment, while their sub-cellular localization were not altered. Taken together, we demonstrated that excessive gallic acid downregulated the expression of auxin efflux *PIN1*, *PIN2, PIN3*, and *PIN7*, leading to reduced auxin accumulation in root tips and inhibiting primary root elongation.

Meanwhile, we monitored the expression of auxin biosynthesis genes in roots when treated without or with 100 μM, 200 μM, and 500 μM gallic acid. Our qRT-PCR data showed that most of the genes examined exhibited similar expression levels in gallic acid-treated and untreated roots (**Supplementary Figure S8**), implying that *in situ* auxin biosynthesis in roots may not be affected by excessive gallic acid.

### Triple mutant *pin1 pin3 pin7* exhibits reduced sensitivity to gallic acid

**Figure 4:**
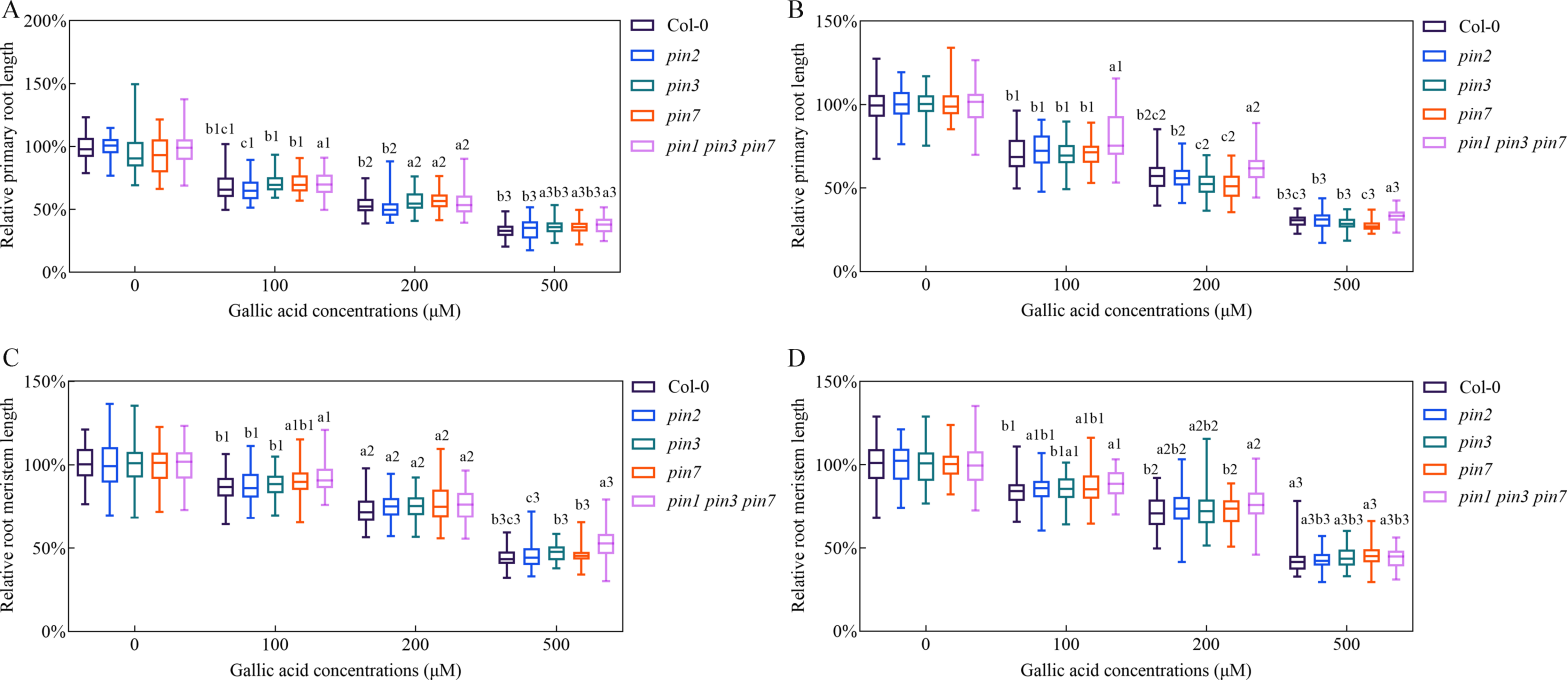
Triple mutant *pin1 pin3 pin7* was less sensitive in response to gallic acid treatment. **(A, B)** The relative lengths of primary roots in wild-type plants, *pin2*, *pin3*, *pin7*, and *pin1 pin3 pin7* treated with 0 μM, 100 μM, 200 μM, and 500 μM gallic acid for 5 d **(A)** and 7 d **(B)**. **(C, D)** The relative lengths of meristem zones in wild-type plants, *pin2*, *pin3*, *pin7*, and *pin1 pin3 pin7* treated with 0 μM, 100 μM, 200 μM, and 500 μM gallic acid for 5 d **(C)** and 7 d **(D)**. Identical numbers following letters represent statistical data on the same gallic acid concentration, and different letters indicate significantly different values (Woller-Duncan test, *P*<0.05).

To further reveal the possible involvements of *PIN1*, *PIN2*, *PIN3*, and *PIN7* genes in gallic acid-caused inhibition of primary root growth, we explored the sensitivity of *pin* mutants in response to excessive gallic acid treatment in terms of primary root length and root meristem size. Single mutant *pin2* exhibited similar sensitivity as wild-type plants (**Figure 4**). Although *pin3* and *pin7* single mutants only exhibited a slightly lower sensitivity in comparison to wild-type seedlings, the *pin1 pin3 pin7* triple mutant was even more tolerant to gallic acid than the single mutant, implying an additive role of *PIN1*, *PIN3*, and *PIN7* in the excessive gallic acid-mediated root growth inhibition (**Figure 4**).

### Gallic acid promotes the stability of AXR3/IAA17 protein

**Figure 5:**
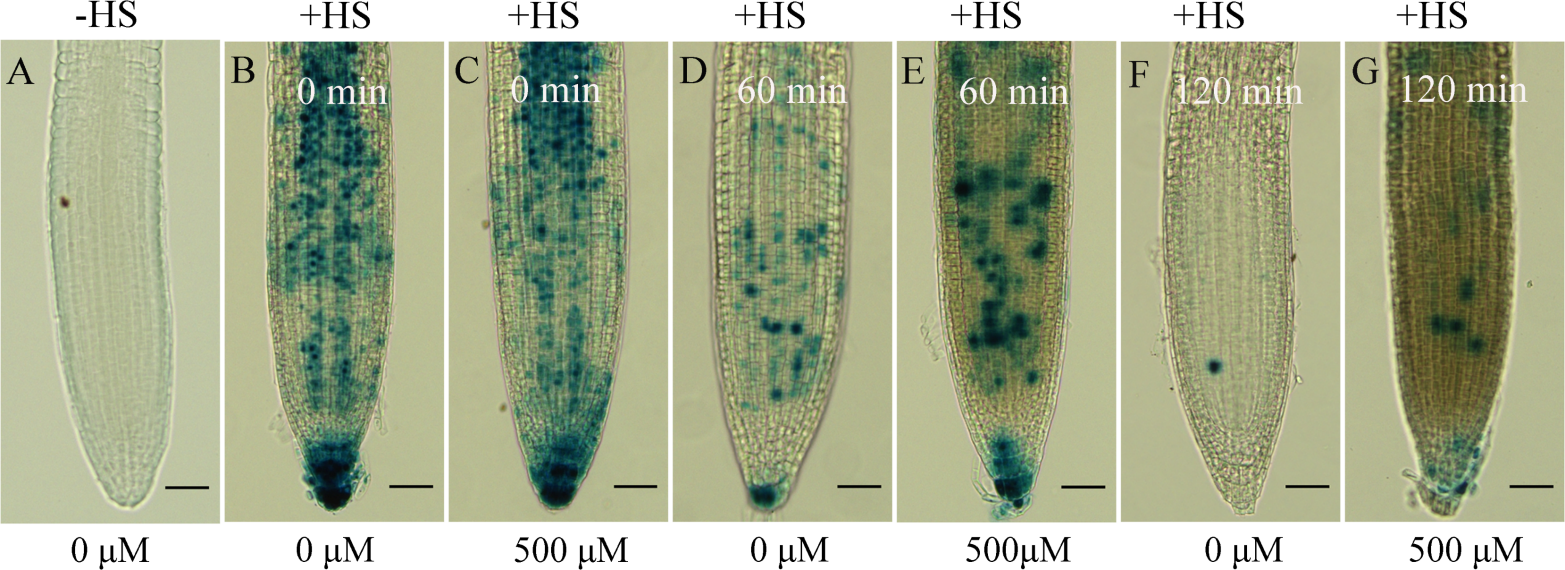
Gallic acid enhances the stability of IAA17 protein. GUS histochemical staining of *HS::AXR3NT-GUS*. Five-d-old seedlings were heat shocked at 37˚C for 2 h in dark and treated without or with 500 μM gallic acid for 0 min, 60 min, or 120 min at 23˚C, followed by GUS staining. HS, heat shock. Bars=50 μm.

Then, we wondered whether auxin signaling was affected by excessive gallic acid. Auxin-induced AUX/IAA protein degradation is generally considered to be a key process in auxin signaling (Lakehal et al., 2019; Liu et al., 2015; Overvoorde et al., 2010). The *HS::AXR3NT-GUS* transgenic line was constructed by fusing the amino terminus (NT) domain II of *AXR3* with *GUS* gene, under the control of a heat shock (HS) inducible promoter (Gray et al., 2001). Therefore, we used the transgenic line *HS::AXR3NT-GUS* to explore whether gallic acid treatment directly affects the protein stability of AXR3/IAA17. We found that without heat shock, the AXR3NT-GUS fusion protein was barely detected (**Figure 5A**), and at the beginning of gallic acid treatment, similar amounts of AXR3NT-GUS fusion protein were accumulated as indicated by GUS staining (**Figures 5B, C**). Moreover, GUS activity in the roots of *HS::AXR3NT-GUS* under 500 μM gallic acid treatment was significantly higher than that in those control roots at both 60 min and 120 min (**Figures 5D-G**), indicating that gallic acid treatment promotes the stability of AXR3 protein.

### Auxin signaling mutant *tir1 afb2 afb3* and *axr3-3* are less sensitive to gallic acid

**Figure 6:**
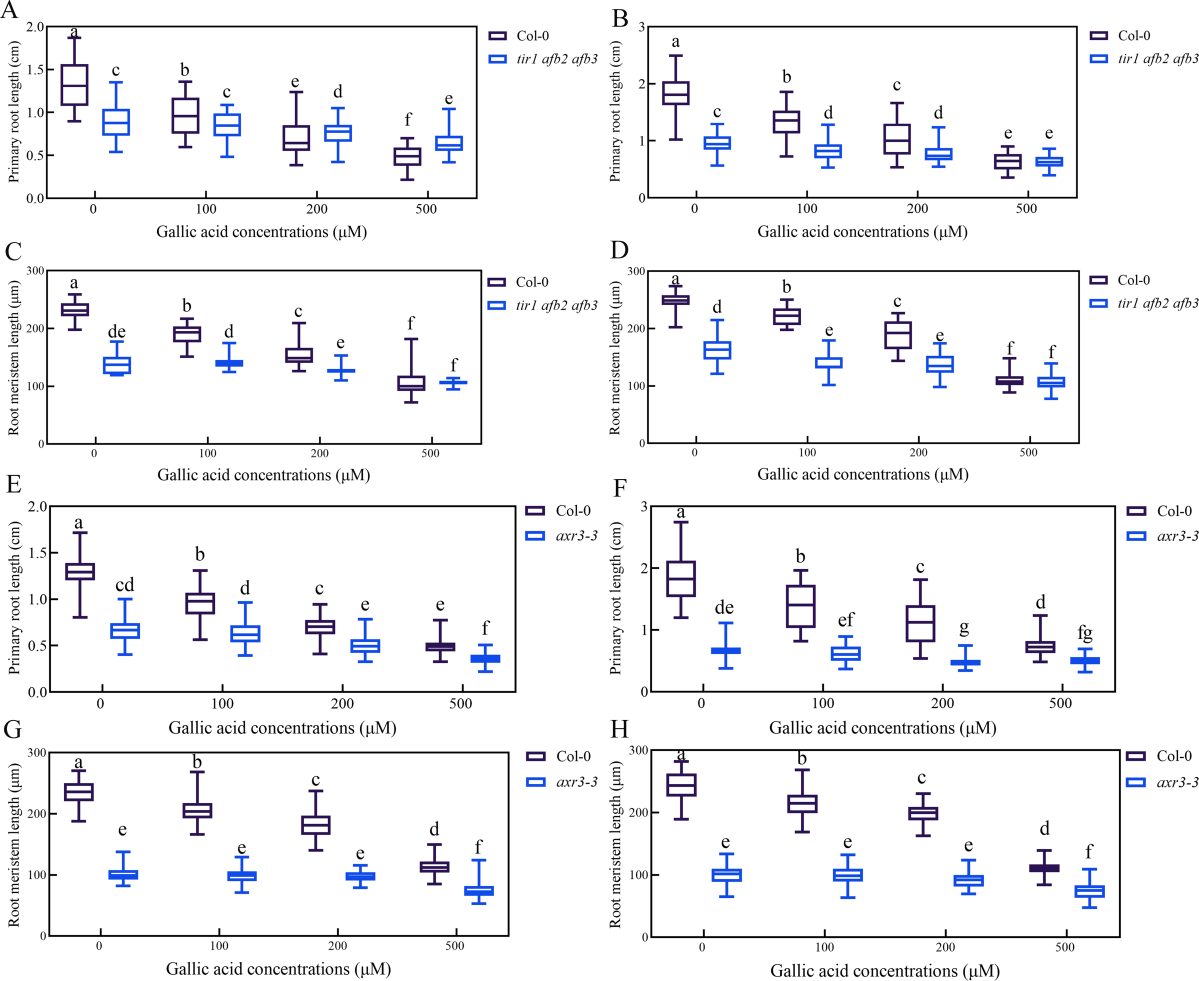
Auxin signaling mutant *tir1 afb2 afb3* and *axr3-3* are less sensitive to gallic acid. **(A, B)** The lengths of primary roots in wild-type plants and *tir1 afb2 afb3* treated with 0 μM, 100 μM, 200 μM, and 500 μM gallic acid for 5 d **(A)** and 7 d **(B)**. **(C, D)** The lengths of meristem zones in wild-type plants and *tir1 afb2 afb3* treated with 0 μM, 100 μM, 200 μM, and 500 μM gallic acid for 5 d **(C)** and 7 d **(D)**. **(E, F)** The lengths of primary roots in wild-type plants and *axr3-3* treated with 0 μM, 100 μM, 200 μM, and 500 μM gallic acid for 5 d **(E)** and 7 d **(F)**. **(G, H)** The lengths of meristem zones in wild-type plants and *axr3-3* treated with 0 μM, 100 μM, 200 μM, and 500 μM gallic acid for 5 d **(G)** and 7 d **(H)**. Different letters indicate significantly different values (Woller-Duncan test, *P*<0.05).

To explore whether auxin signaling is involved in gallic acid-mediated inhibition of primary root growth, the sensitivity of auxin-related mutants in response to gallic acid treatment was analyzed. Previous studies have shown that auxin signaling mutant *tir1 afb2 afb3* had significantly shorter primary roots and meristem size compared with wild-type plants (Qi et al., 2022; Yu et al., 2022). Our data further revealed that the reduced primary root length and root meristem size in *tir1 afb2 afb3* mutant was not further exacerbated before and after gallic acid treatment (**Supplementary Figures 6A-D**). Meanwhile, *axr3-3*, a gain-of-function mutant of *AXR3/IAA17* with shortened primary roots and root meristem size, also had reduced sensitivity to exogenous gallic acid treatment (**Figures 6E-H**). Loss-of-function mutant *axr3/iaa17* displayed similar primary root growth inhibition as wild-type plants (**Supplementary Figure S9**), probably due to functional redundancy between *AUX/IAA* family genes. Taken together, these results indicated that gallic acid inhibited primary root and meristem elongation by affecting auxin signaling.

## Discussion

As one of the most common secondary metabolites in plants, polyphenols play important roles in regulating plant ecological defense, stress resistance, tissue culture and seedling rooting, and plant flowering (Sharma et al., 2019). Between those common polyphenols in plants, gallic acid has been widely concerned in the fields of antibacterial, anti-inflammatory and anti-cancer (Wu et al., 2024), but its effects in regulating plant root growth and development remains unclear. In this study, we reported that gallic acid had a dual effect of “low promotion and high inhibition” on the elongation of primary root and meristem. While plant root growth was enhanced under lower concentrations of gallic acid, excessive gallic acid could significantly inhibit the elongation of primary root and the cell division activity in root apical meristem (**Figure 1**, **Supplementary Figure S1**). This dual role of gallic acid in regulating plant growth mimics the effects of plant hormones, such as auxin and strigolactone (Ruyter-Spira et al., 2011). Thus, we further analyzed the relationship between gallic acid and auxin, the key regulator of early seedling development in Arabidopsis (Adamowski and Friml, 2015; Yu et al., 2022), and found that excessive gallic acid-caused inhibition of primary root elongation involves *PINs*-mediated auxin accumulation in root tips and *AXR3/IAA17*-mediated auxin signaling transduction.

As one of the most important plant hormones, auxin plays an essential role in plant growth and development (Casanova-Sáez et al., 2021; Rahman, 2013; Zhang et al., 2023). In roots, an auxin gradient in the root tips is required for normal plant root development (Benková et al., 2003). Here, we found that excessive gallic acid reduces auxin contents in roots as suggested by the *DR5::GFP* marker line (**Figures 2A, B**) and confirmed by LC-MS assay (**Figure 2C**). Additional application of IAA partially rescued the reduction of primary root and root meristem (**Figures 2D, E**). This data suggested that auxin is required for gallic acid-mediated inhibition of primary root growth. It is common that environmental cues and endogenous signals modulate auxin contents to coordinate plant growth and development (Dello Ioio et al., 2008; Liu et al., 2015). Besides, recent researches also reported the relationship between auxin-mediated root growth and some secondary metabolites like terpenoids (Araniti et al., 2017; Li et al., 2019). Here, we further established the correlation between plant hormone auxin with gallic acid, one of most abundant polyphenols in plants.

Previous researches have indicated that during the early stage of seedling development in Arabidopsis, shoot-to-root auxin transport play a fundamental role in maintaining auxin contents in root tips and promoting the elongation of young roots (Friml et al., 2003; Wisniewska et al., 2006). Disturbing PAT by exogenous application of NPA or endogenous mutation of auxin transporters often lead to a decreased auxin accumulation in root tips and suppressed primary root elongation (Blilou et al., 2005; Liu et al., 2015; Sauer and Kleine-Vehn, 2019; Su et al., 2022). In this study, we found that gallic acid did not affect the expression of auxin biosynthesis genes in roots (**Supplementary Figure S8**), implying that auxin biosynthesis in roots was not involved in gallic acid-modulated root growth. However, the expression of *PIN1*, *PIN3*, and *PIN7* were significantly down-regulated both at the mRNA and protein levels (**Figures 3C-L**), indicating that gallic acid-caused primary root growth inhibition may involve PINs-mediated PAT. Further observations that the inhibition effects of gallic acid on primary root growth was compromised in the presence of NPA (**Figures 3A, B**) and that the *pin1 pin3 pin7* triple mutant exhibited reduced sensitivity to excessive gallic acid treatment (**Figures 4A-D**) clearly suggested that gallic acid suppresses primary root growth through modulating auxin transport. We noted that although the *pin3* and *pin7* single mutants only had a weak phenotype, the *pin1 pin3 pin7* triple mutant exhibited a stronger less-sensitive phenotype than the single mutants. These results implied that *PIN1*, *PIN3*, and *PIN7* function additively to mediate gallic acid-derived root growth regulation. Given that *PIN1*, *PIN3*, and *PIN7* display overlapping expression patterns in root vascular tissue and are responsible for shoot-to-root polar auxin transport (Dello Ioio et al., 2008), the functional redundancy between them ensures normal auxin transport when some of the genes loss function, and thus enhances root developmental plasticity. The fact that NAA had a better recovery effect than IAA on primary root length and root meristem size (**Figures 2D, E**, **Supplementary Figure S7**) also indirectly indicated the role of PIN efflux facilitators in excessive gallic acid-induced primary root growth in Arabidopsis.

Auxin signaling is also critical for plant growth and development (Leyser, 2018; Yu et al., 2022). Alteration of auxin receptors or downstream suppressors and transcription factors lead to plant growth deficiency. Auxin receptor mutant *tir1 afb2 afb3* had a reduced primary root and was less sensitive to IAA (Dharmasiri et al., 2005; Qi et al., 2022). Gain-of-function mutant *axr3-3*, harboring a single point mutation (V89G) in domain II and stabilized IAA17 protein, had a severe root deficiency, including much shortened primary root and root meristem size, loss of gravitropism (Rouse et al., 1998). We further found that gallic acid promotes the protein stability of AXR3/IAA17 as indicated by *HS::AXRNT-GUS* reporter line (**Figure 5**). Meanwhile, both *tir1 afb2 afb3* and *axr3-3* were insensitive to gallic acid treatment in terms of primary root length and root meristem size (**Figure 6**). We noted that additional application of IAA (**Figures 2D, E**) or NAA (**Supplementary Figure S7**) only slightly rescued root growth deficiency under gallic acid treatment. Also, the *pins* mutants (**Figure 4**) only exhibited a slightly insensitive to gallic acid treatment. But the auxin signaling mutants *tir1 afb2 afb3* and *axr3-3* both had strong phenotypes, since they were both insensitive to gallic acid treatment compared with wild-type plants (**Figure 6**). Given that gallic acid could facilitate the protein stability of AUX/IAA protein like AXR3/IAA17, the weak phenotype of auxin recovery assays and *pins* mutants might be due to the inhibition effect of gallic acid on auxin signaling. Thus, we demonstrated that auxin signaling is also involved in gallic acid-suppressed primary root growth.

Cellular redox status is fundamental for cellular functions, and maintained by the balance between series of antioxidant systems and generation of ROS (Ogawa et al., 2016). Although polyphenols had long been recognized as natural scavengers of ROS and were reported to be involved in modulating plant growth and development (Sharma et al., 2019), the link between polyphenols with plant hormones (auxin in particular) was largely missing. Previously, antioxidants like GSH were reported to have an influence on modulating auxin-mediated growth and development (Koprivova et al., 2010). Here, we reported that the natural polyphenol antioxidant gallic acid regulated auxin transport and signaling and thus modulated primary root elongation in Arabidopsis. The observation that 3,4-dihydroxybenzoic acid (the analog of gallic acid or 3,4,5-trihydroxybenzoic acid) had a slightly weaker inhibitory effect on primary root elongation as gallic acid (**Figures 1A, B**, **Supplementary Figure S3**) further suggested that polyphenols have an influence on plant root growth. Thus, our data provided experimental evidence for the correlation between polyphenols with auxin and established a model for polyphenols-mediated regulation of plant growth.

In conclusion, we demonstrated that gallic acid play a pivotal role in regulating root growth and development possible through modulating PINs-mediated auxin transport and AXR3/IAA17-mediated auxin signal transduction in Arabidopsis. These results provide new insights into the role of the secondary metabolite gallic acid in plants.

## Data Availability

The datasets presented in this study can be found in online repositories. The names of the repository/repositories and accession number(s) can be found in the article/**Supplementary material**.
