# Supplementary material for: Gallic acid regulates primary root elongation via modulating auxin transport and signal transduction
Source: Front Plant Sci. 2024 Sep 2;15:1464053. doi: 10.3389/fpls.2024.1464053 (PMC11402708; doi:10.3389/fpls.2024.1464053)
Supplement: Supplementary file 1 [file DataSheet1.docx]

Supplementary Material


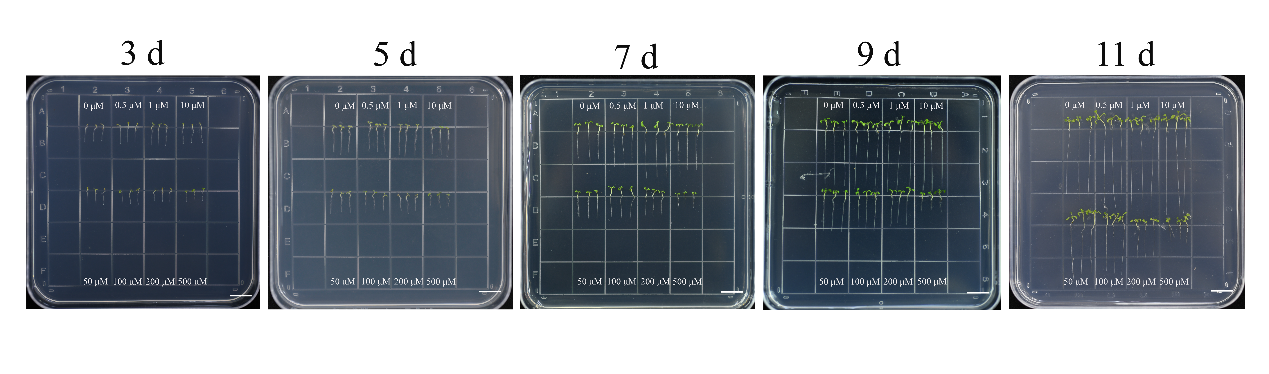


**Supplemental Figure S1**. Root growth phenotype of wild-type seedlings under different concentrations of gallic acid. The seedlings were grown in separate plates and transferred to a fresh plate for picture taking at 3 dpg, 5 dpg, 7 dpg, 9 dpg, and 11 dpg, respectively. Bars = 1 cm.


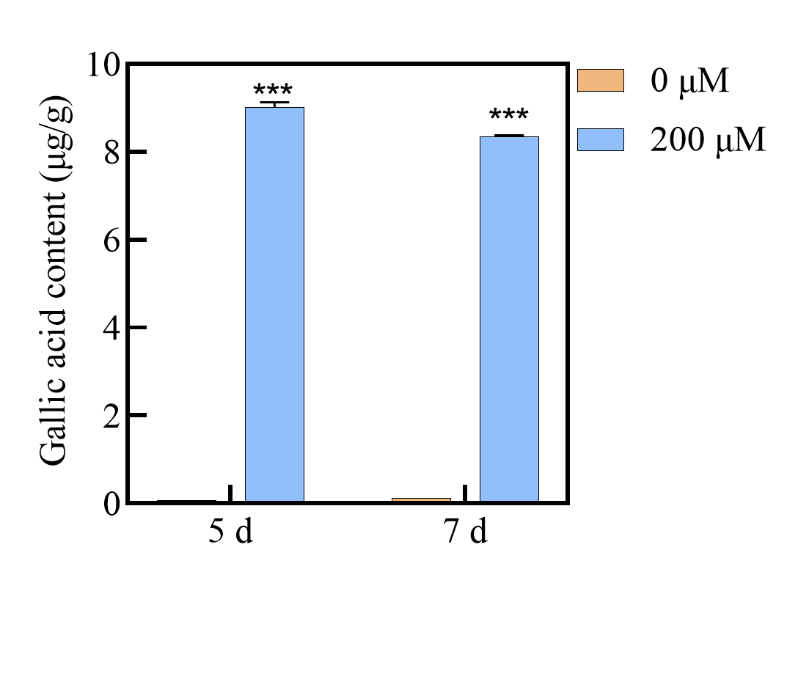


**Supplemental Figure S2**. Quantification of gallic acid contents in Arabidopsis. LC-MS analysis of gallic acid contents in wild-type Arabidopsis seedlings grown on 1/2 MS plates supplied without or with 200 gallic acid for 5 d and 7 d. Error bars represent SE. Asterisks indicate significant differences with respect to the corresponding control (Student’s *t* test, ****P*<0.001).


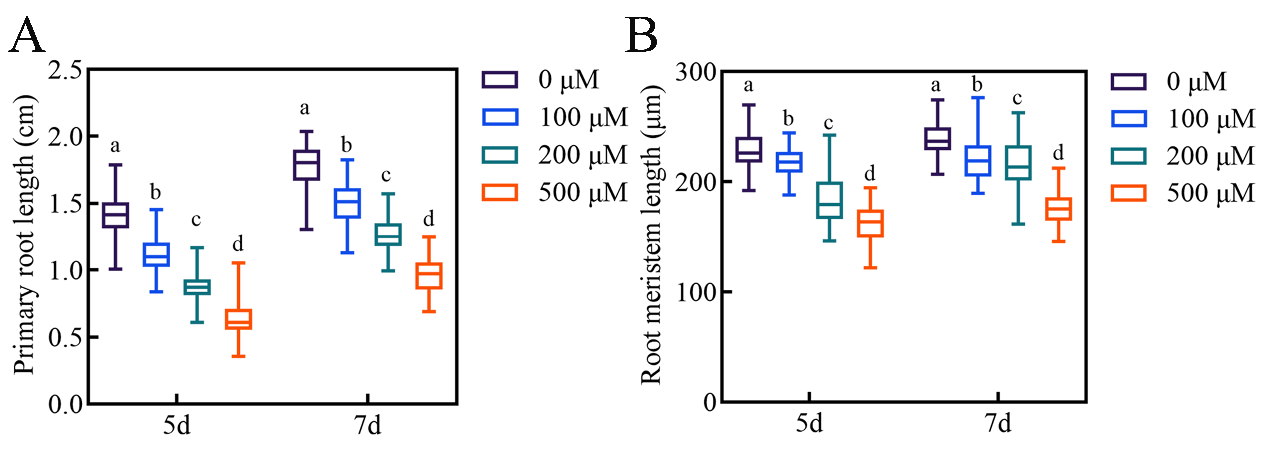


**Supplemental Figure S3**. The effects of 3,4-dihydroxybenzoic acid on primary root elongation. Primary root length (A) and root meristem length (B) of wild-type plants treated with 0 μM, 100 μM, 200 μM, and 500 μM 3,4-dihydroxybenzoic acid for 5 d and 7 d. Different letters indicate significantly different values (Woller-Duncan test, *P*<0.05).

**
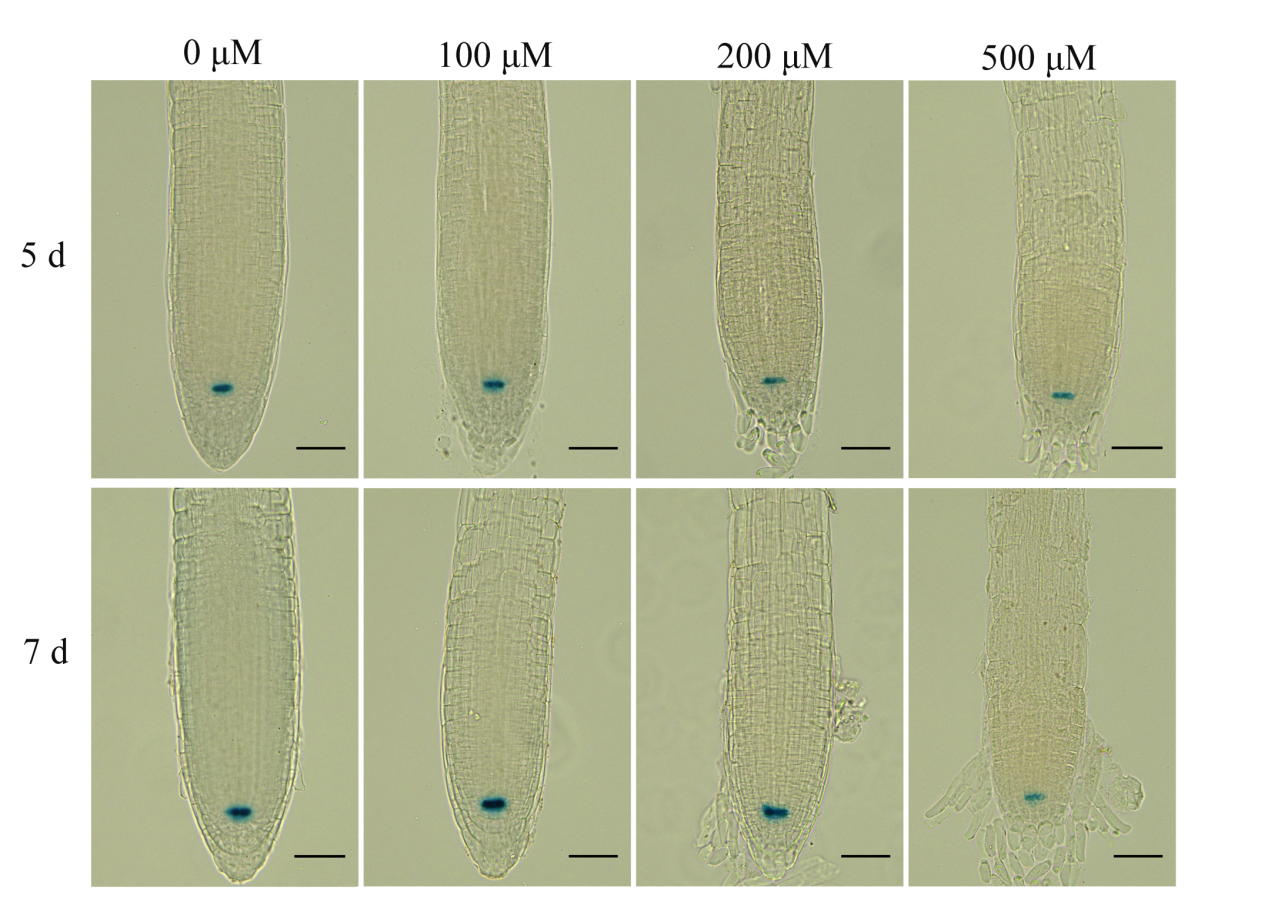
**

**Supplemental Figure S4**. The effects of excessive gallic acid on the expression of *QC25::GUS*. GUS histochemical staining of *QC25::GUS* reporter line treated with 0 μM, 100 μM, 200 μM, and 500 μM gallic acid for 5 d and 7 d. Bars = 50 μm.

**
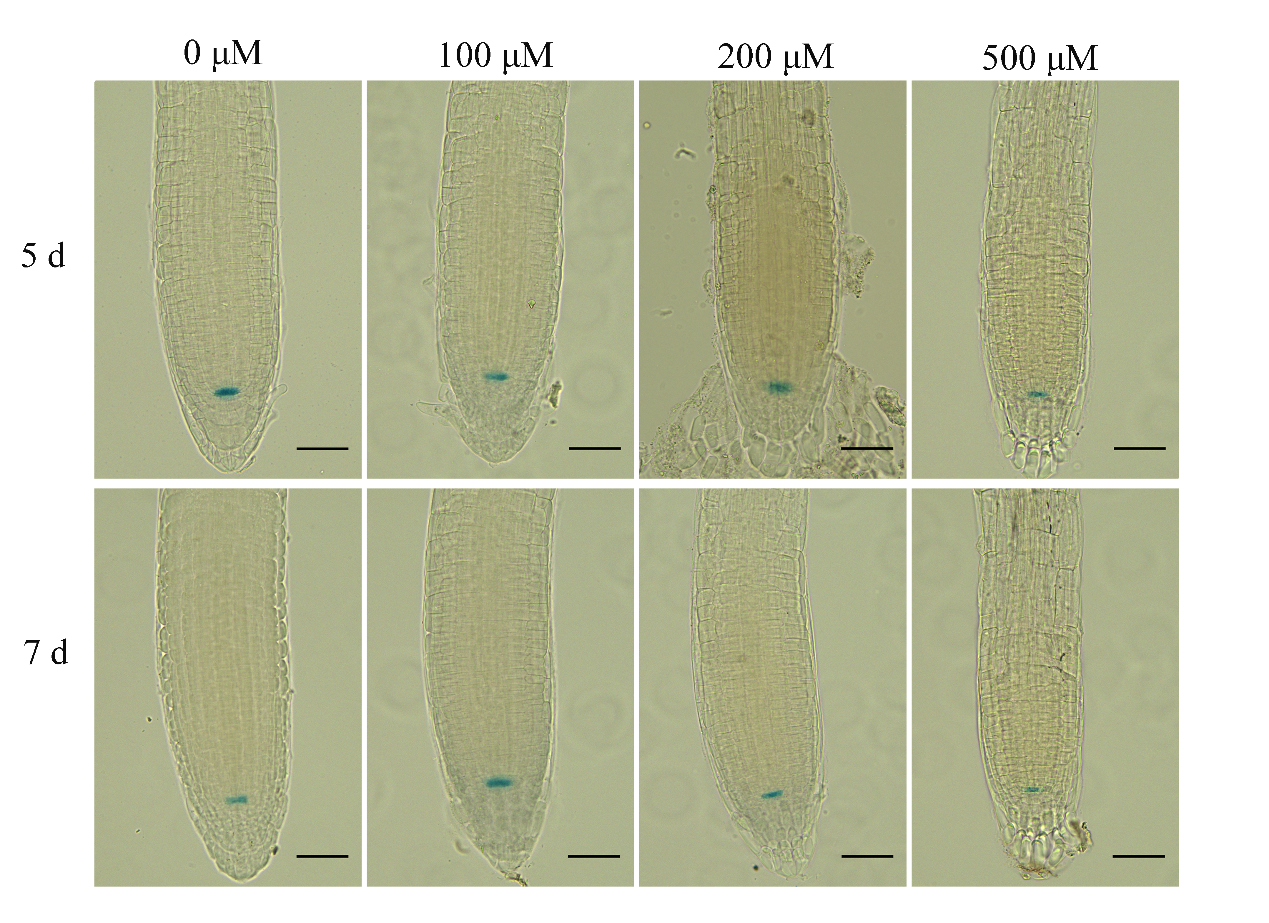
**

**Supplemental Figure S5**. The effects of excessive gallic acid on the expression of *QC46::GUS*. GUS histochemical staining of *QC46::GUS* reporter line treated with 0 μM, 100 μM, 200 μM, and 500 μM gallic acid for 5 d and 7 d. Bars = 50 μm.


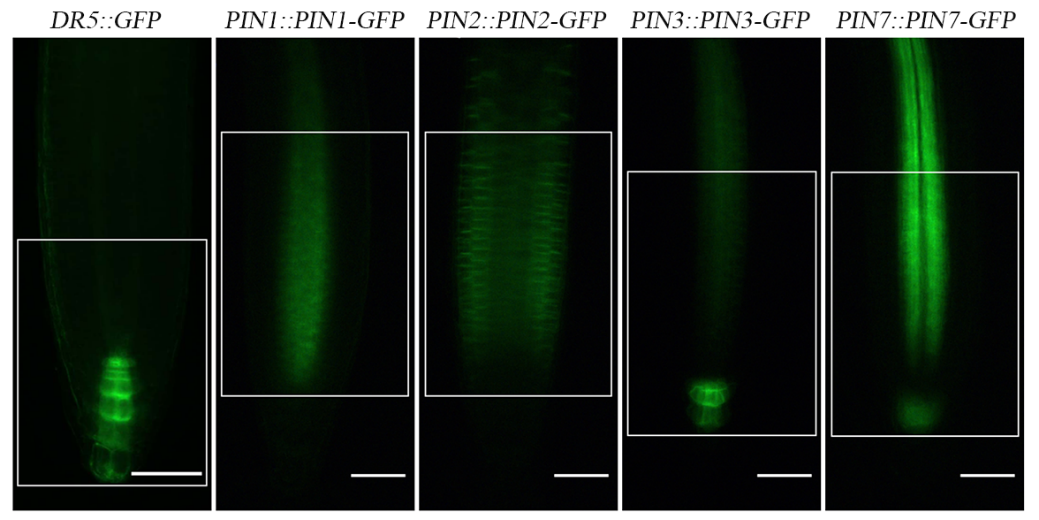


**Supplemental Figure S6**. The regions of the root tips for quantifying the fluorescence signal. Bars=50 μm.


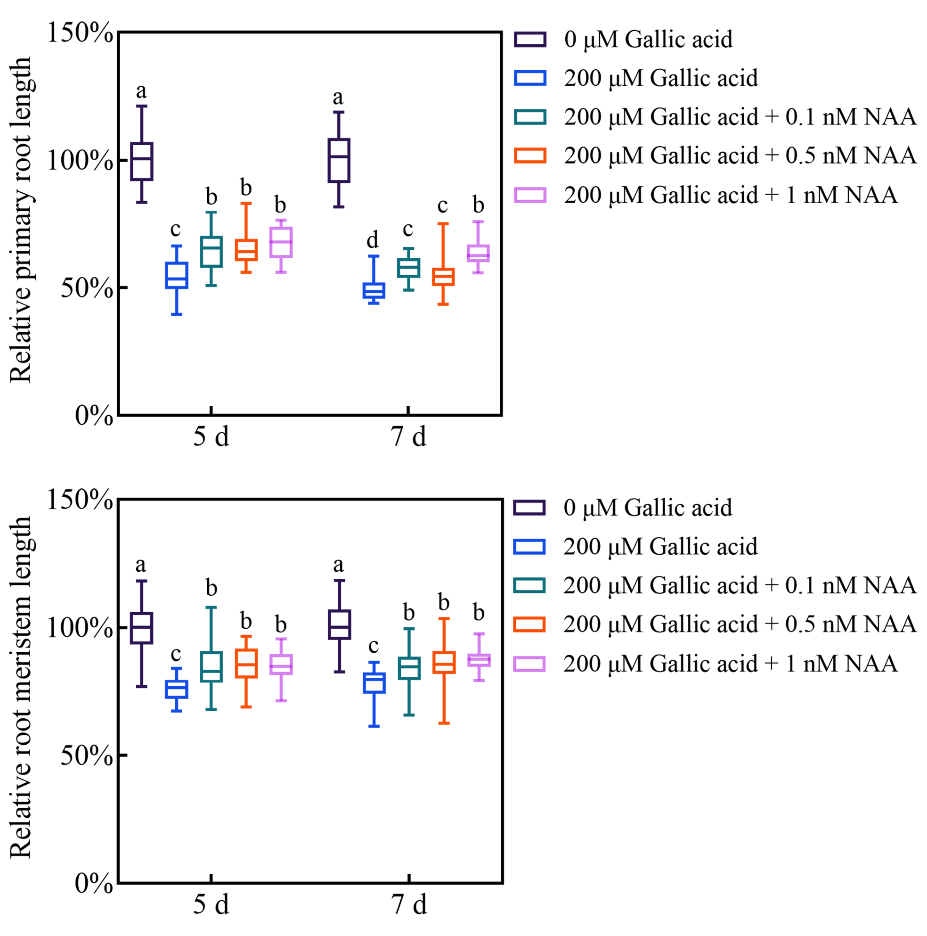


**Supplemental Figure S7**. NAA partially rescued root growth inhibition under excessive gallic acid treatment. **A** and **B** The relative lengths of primary roots (A) and meristem zones (B) in wild-type plants treated without or with 200 μM gallic acid plus 0 nM, 0.1 nM, 0.5 nM or 1 nM NAA for 5 d and 7 d. Different letters indicate significantly different values (Woller-Duncan test, *P*<0.05).

**
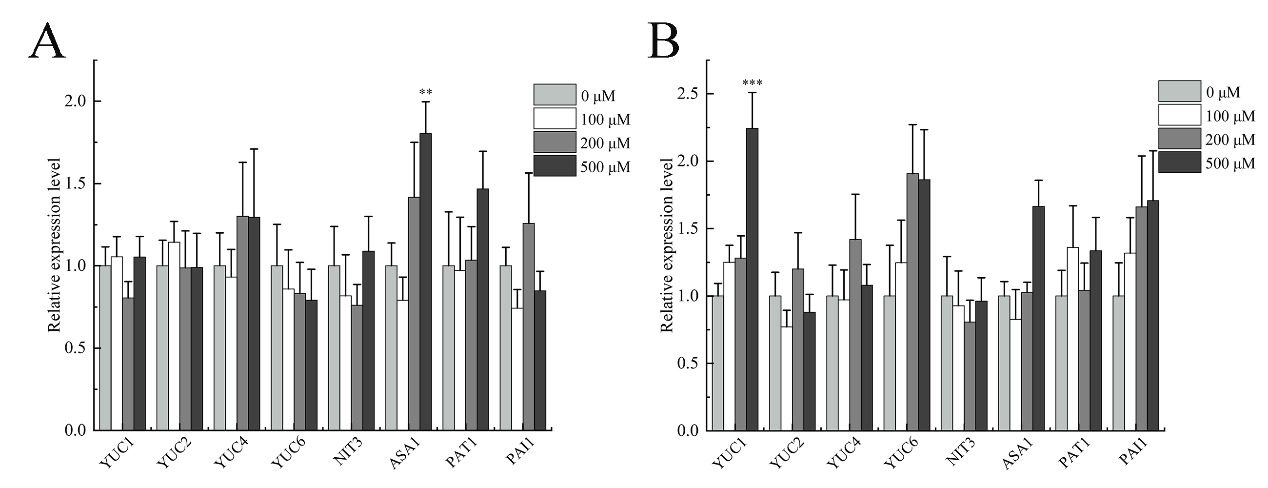
**

**Supplemental Figure S8**. The expression levels of auxin biosynthesis genes under gallic acid treatment. **A** and **B** The relative expression levels of auxin biosynthesis genes in wild-type roots treated with 0 μM, 100 μM, 200 μM, and 500 μM gallic acid for 5 d (A) and 7 d (B). The expression levels of the indicated genes in untreated roots were set to 100%. Error bars represent SE, and asterisks indicate significant differences with respect to the corresponding control (Student’s *t* test, ***P*<0.01, ****P*<0.001).


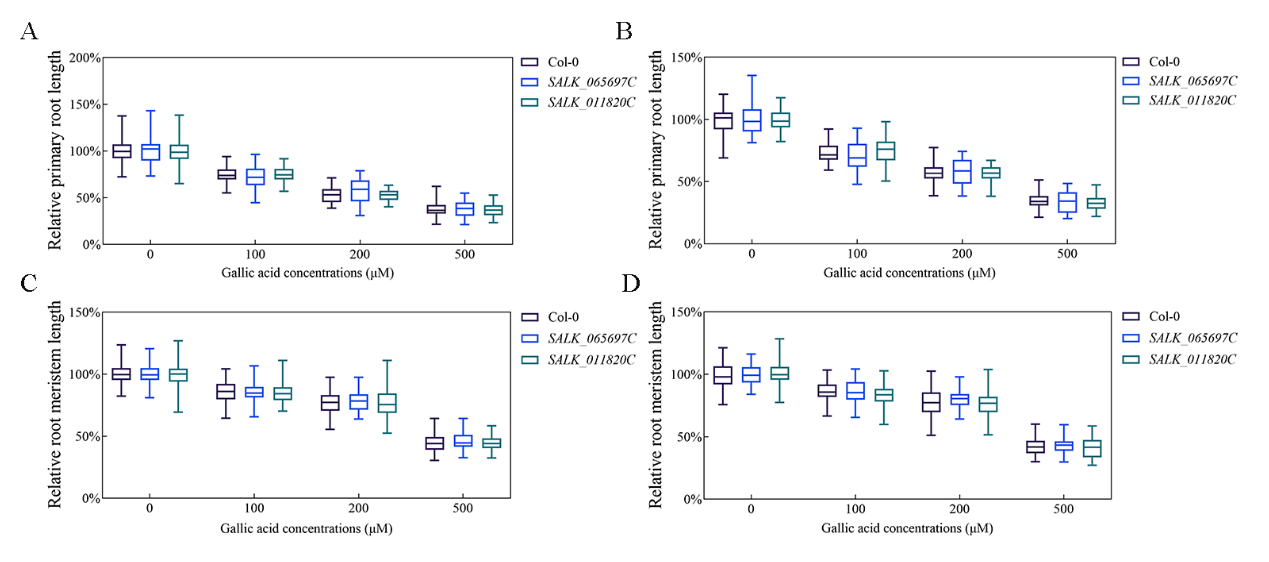


**Supplemental Figure S9**. The sensitivity of *axr3/iaa17* loss-of-function mutant (*SALK_065697C* and *SALK_011820C*) in response to gallic acid treatment. **A** and **B** The relative lengths of primary roots in WT, *SALK_065697C*, and *SALK_011820C* treated with 0 μM, 100 μM, 200 μM, and 500 μM gallic acid for 5 d (A) and 7 d (B). **C** and **D** The relative lengths of meristem zones in WT, *SALK_065697C*, and *SALK_011820C* treated with 0 μM, 100 μM, 200 μM, and 500 μM gallic acid for 5 d (C) and 7 d (D).

**Supplemental Table S1.** List of primers used in this paper.

**Primer name Sequence (5’ to 3’)**

*qeIF4A* F TCATAGATCTGGTCCTTGAAACC

*qeIF4A* R GGCAGTCTCTTCGTGCTGAC

*qPIN1* F GGAGACTTAAGTAGGAGCTCAGCA

*qPIN1* R CCAAAAGAGGAAACACGAATG

*qPIN3* F TCTTTGATTAGGTTCGGGTAACTC

*qPIN3* R GCTCATGTGAAACTGGAACAAG

*qPIN7* F CCAAGATTAGTGGAACGCAAC

*qPIN7* R GAAAAGGGTTTTTGGATCCTC

*q*YUC1 F TGGAGAGTAAAGACTCATGAT

*q*YUC1 R GTACTCACTCGCGTGAACGAT

*q*YUC2 F GGTGACACGGATCGGTTAGGGT

*q*YUC2 R TGCCGAATAATGCATTACCCGT

*q*YUC4 F TGGAGGTCAGCTTGGATCTT

*q*YUC4 R TTCCAAATGTTGATAGACCAAAAA

*q*YUC6 F CTCGTTGTCAGAGACGCTGT

*q*YUC6 R AACCAAAAGGAAACGGTCAA

*q*NIT3 F AGCGAAGTTGGTGTTGTTTCCC

*q*NIT3 R CCAACTCAGCCAATCTTTCCAC

*q*PAI1 F CATCAGCCAGAGATGCAGCTA

*q*PAI1 R CAGAGGAATCAGCTGCTCTCA

*q*ASA1 F GTAGAGAAGCTTATGAACATCGA

*q*ASA1 R GGTGCACCACTAACTGTTCCCAC

*q*PAT1 F ATGGTTATTGCGGTGGCGACGA

*q*PAT1 R ATCGTCGCCGACTCAATGTCGG
